# Development of a self-report instrument for measuring in-class student engagement reveals that pretending to engage is a significant unrecognized problem

**DOI:** 10.1371/journal.pone.0205828

**Published:** 2018-10-17

**Authors:** Kathryn A. Fuller, Nilushi S. Karunaratne, Som Naidu, Betty Exintaris, Jennifer L. Short, Michael D. Wolcott, Scott Singleton, Paul J. White

**Affiliations:** 1 Eshelman School of Pharmacy, University of North Carolina, Chapel Hill, North Carolina, United States of America; 2 Faculty of Pharmacy and Pharmaceutical Sciences, Monash University, Melbourne, Victoria, Australia; Kyoto University, JAPAN

## Abstract

Student engagement during classes includes behavioural, cognitive and emotional components, and is a pre-requisite for successful active learning environments. A novel approach to measuring student engagement was developed, involving triangulation of real-time student-self report, observation by trained observers and heart rate measurement. The self-report instrument was evaluated in four separate cohorts (n = 123) at Monash University and the University of North Carolina. The six item self-report demonstrated good reliability (Cronbach’s alpha values ranged from 0.7–0.81). The self-report showed predictive validity in that small group activities were rated as significantly more engaging than didactic lecturing. Additionally, there was significant inter-instructor variability and within-class variability, indicating good discrimination between classroom activities. This self-report may prove useful to academic teaching staff in evaluating and refining their active learning activities. Independent observation was not found to correlate with student self-report, due in part to students who were pretending to engage being rated as engaged by an observer. Strikingly, students reported that they were pretending to engage for 23% of class time, even for highly regarded instructors. Individual participants were rated as engaged for 42 of the 46 intervals for which they reported that they had “pretended to engage”, indicating that the two observers were unable to detect disengagement during periods in which students pretended to engage. Instructors should be aware that student cues such as eye contact and nodding may indicate pretending to engage. One particular self-report item; “*I tried a new approach or way of thinking about the content*”, correlated positively with heart rates, and a controlled study reproduced this finding during two activities that required students to try a new approach to understanding a concept. Agreement with this item also correlated with superior performance on two in-class written assessment tasks (n = 101, p<0.01). Further use of this tool and related educational research may be useful to identify in-class activities that are engaging and likely to lead to improved student attainment of learning outcomes.

## Introduction

Many institutions, including the two pharmacy and pharmaceutical sciences education institutions at which this study was conducted, have transformed their curricula to focus heavily on active learning [1, 2]. Active learning is an instructional approach that involves students as active participants in classes, tackling activities, often in small groups, to gain mastery of learning outcomes by doing rather than by listening. This study focuses on measuring student engagement in the context of active learning; i.e. engagement with an active learning task. Student engagement assumes even greater importance in this context than with traditional lecturing, since engagement with tasks is required for active learning to work. If students engage with a task, they may (or may not) gain desired knowledge or skills. However, students that do not engage will certainly not gain anything from that task. Students may argue that they have the potential to gain the same knowledge or skills through independent study rather than attendance at classes or participation in active learning tasks, of course. However, evidence from many studies shows that active learning approaches are highly effective ways to promote student learning [1–6]. Once the *‘locus of control’* of the class is handed over to the students, the level of engagement with the activities is directly related to student achievement of learning outcomes; “*The core elements of active learning are student activity and engagement in the learning process*” [5]. In the current environment of significant change in how we teach, validated methods are required to provide real-time feedback to instructors that will inform a cycle of quality improvement.

Student engagement is multi-faceted and challenging to define (see [7, 8]). The meta-construct of student engagement involves three major elements: behavioral, emotional and cognitive engagement [9]. Behavioural engagement relates to observable student activity, including how they engage with learning tasks. Emotional engagement relates to how students feel about learning; their level of interest, enjoyment or frustration and dissatisfaction. Cognitive engagement relates to how they think about their learning, including meta-cognitive engagement via strategy use and self-regulation.

Methods to measure student engagement *related to specific tasks* are important to inform a feedback cycle of necessary precision. Valid and reliable instruments that measure global student engagement abound; many were developed for use with K-12 students but have been adapted for use in higher education. These include the Mainstream Code for Instructional Structure and Student Academic Response [MS-CISSAR] [10], Engagement versus Disaffection with Learning [EvsD]) [11], and the Motivation and Engagement Scale [MES] [12]. The Motivated Strategies for Learning Questionnaire (MSLQ) was developed for use with college students [13], as was the National Survey of Student Engagement (NSSE) [14]. Whilst some of these address the cognitive, emotional and behavioural components of engagement, they generally contain large question banks that are not designed to evaluate engagement related to a particular activity. For example, the HSSSE contains 121 items (http://www.indiana.edu/~ceep/hssse/) which ask students to rate engagement for teaching modes (teacher lectures, discussion and debates etc.) rather than specific class activities.

Most existing measures of student engagement are not designed to look at changes during class and do not provide moment-to-moment data. Of those that are directed at real-time engagement, a few examples stand out. Lane and colleagues [15] produced an observational instrument that reliably measured behavioural engagement in large classes. O’Malley et al developed an observation-based instrument to measure in class student engagement, however this instrument did not involve self-report [16]. *‘Experience sampling’*, where students self-report their engagement at intervals, is a highly cited approach used by Shernoff and colleagues [17, 18]. The limitations of using extended self-report instruments have been previously characterized (see Fredricks and McColskey [19]); including the interference with student learning and the difficulty in ensuring student participation. Notably, the primary measure of student engagement used by Shernoff et al (2008) was derived from three items in the 45 item experience sampling method (ESM) instrument: “How well were you concentrating”; “Did you find the activity interesting”; and “Did you enjoy what you were doing?”. This suggested to us that a shorter self-report instrument that addressed behavioural, cognitive and emotional engagement with a small number of items might provide a robust engagement measure in real-time without disrupting learning.

Engagement with active learning approaches can help students attain skills as well as knowledge, including communication or critical thinking skills. We recently found that students who engage with an active learning approach do much better than less engaged peers on higher order examination questions requiring solution of novel problems, but not on lower order knowledge questions [20]. Since there are many key factors that affect engagement within the classroom environment, this study focuses on combining methods of measurement to determine the factors that contribute most positively to the level of student engagement. Therefore, the purpose of this current study was to produce, apply and validate a new triangulation method for measuring student engagement during active learning class sessions.

Heart rate measurement was included in the study in order to look for positive or negative task-related emotions. A large body of work has established a range of task-related emotions (joy, boredom, satisfaction, hopelessness, frustration, shame, pride) that can occur prior to tackling a task (prospective emotions), during a task or following a task. These emotions, over time, affect student motivation for future tasks and therefore affect student engagement [21]. “*Appraisals of control and values are essential to arousal of activity-related emotions (enjoyment*, *frustration*, *boredom) as well as outcome-related emotions (joy*, *pride*, *shame)* [22]. Heart rate rises during both positive and negative emotions due to limbic system-derived sympathoadrenal activation, and thus may provide an objective measure of emotional engagement that could be correlated with observed or self-reported engagement. Correlation of self-report items addressing positive or negative task-related emotions with heart rate was included in this study in an attempt to reveal any associations that affected student engagement. The new triangulation method measured student engagement in three ways: (1) self-report at intervals; (2) empirical observation by a trained observer; and (3) heart rate. Student self-reported engagement (shortened for feasibility) and observer rating of engagement were included as these have been the mainstay of previous methods to measure engagement as described above. Heart rate was added as a potentially novel method of evaluating a physiological outcome of engagement. It is hypothesized that using this new triangulation method of measuring student engagement could provide a more robust, reliable metric to guide educators in the design of effective active learning activities. A real-time triangulating method would provide sufficient precision to determine whether in class approaches are engaging to students and inform future teaching.

We asserted that the triangulation of task-related student engagement would show the following characteristics: reliability of the self-report as assessed via Cronbach’s alpha, face validity as assessed during student focus groups, and construct validity in that the instrument would discriminate between instructors and between passive experiences and active learning activities. We predicted that heart rate would correlate with self-reported positive and negative emotional engagement. We predicted that the engagement triangulation method would predict student performance on an assessment related to a specific task for which engagement had been evaluated.

## Methods

### Participants

The participants of the study consisted of Pharmacy and Pharmaceutical Science students from Monash University in Melbourne, Australia, and Pharmacy students from the University of North Carolina Eshelman School of Pharmacy in Chapel Hill, North Carolina. The study was approved by the Monash University Human Research Ethics Committee and the University of North Carolina Institutional Review Board.

### Participant information and study design

Participants were recruited via announcements in class, and written informed consent was obtained during a subsequent session. 87 female students and 36 male students with median age 21 years participated in the study. The recruitment approach used may have resulted in biased sampling in that more engaged students may have been more likely to participate in the study. We noted that the GPA of cohorts one and two was not significantly different to their respective cohort GPA, however this does not rule out a sampling bias.

Four cohorts were used (see Table 1 for summary). In the first three cohorts we evaluated engagement during real classes using the self-report, observation and heart rate measurement (Part 1, described below). The second cohort was also involved in a controlled experiment to attempt to reproduce, in controlled conditions, a relationship between engagement and heart rate that had emerged during analysis of the first two cohorts (Part 2, described below). Finally, a fourth cohort was used to test the ability of the self-report instrument to predict student performance on an assessment related to a specific task for which engagement had been evaluated (Part 3, described below).

**Table 1:** The four cohorts of students and the parts of the study in which they participated.

Cohort	# students	# classes (# activities)	Self-report	Observation	Heart rate	Controlled experiment	Written task
**1. Monash 1**^**st**^ **year Pharmaceutical Science**	9	8 (40)	Yes	Yes	Yes	No	No
**2. Monash 2**^**nd**^ **year Pharmacy**	8	4 (20)	Yes	Yes	Yes	Yes	No
**3. University of North Carolina**	5	9 (45)	Yes	Yes	Yes	No	No
**4. Monash 1**^**st**^ **year Pharmacy**	101	1 (2)	Yes	No	No	No	Yes

Five instructors were involved in the study. There were four male and one female instructors, all experienced in teaching at University level. The instructors had a mean of 18 years teaching experience (range 10–20), 4.8 years teaching the topic investigated (range 2–10), and 4 teaching awards of any type (range 0–10).

#### Part 1. In-class sessions to test instrument validity and reliability (cohorts one–three)

The three variables used to rate engagement, namely student self-report, observer rating and heart rate, were measured for each of the four cohorts, in order to test the validity and reliability of each of the variables. Across the four cohorts, in 22 one-hour class sessions, 123 students completed the engagement self-report for 107 activities; at the same time they were rated for engagement by observation using a spreadsheet, and their heart rate was measured using a wristband heart rate monitor supplied to them by the investigators. Variables predicted to affect student engagement based on previous literature were used as measures of construct validity; these were the level Bloom’s cognitive domain of the activity (as previously reported; [1]), the extent of active learning involved, and the instructor. We measured the extent of active learning using the ordinal rating scale that we developed and validated. This scale consisted of: didactic; socratic, small-group closed activities and small group open activities. We treated each instructor as a nominal variable, and looked for variability in engagement between instructors and between activities for a given instructor as an indicator of the ability of the instrument to discriminate between activities.

#### Part 2. Controlled experiment (cohort two)

Seven students from cohort two participated in a controlled experiment to determine the effects of activities that had been pre-determined to be interesting or not interesting; active or passive; and promoting novel thinking or not promoting novel thinking (independent variables) on heart rate (dependent variable). The controlled experiment involved five activities in total. For each activity, heart rate prior to the activity was measured for 30 seconds to establish a baseline. The activities were conducted for four minutes, during which heart rate was measured, after which a final 30 seconds was allowed to elapse prior to the next activity.

Three activities were pre-designed by students:

- Participants were asked to find a video that they could predict would be boring to all participants: participants chose to watch a flipped class pre-recording that they had found in the past to be boring, this task was designed to determine a baseline heart rate;
- Participants were asked to find a video that they could predict would be interesting to all participants: participants chose to watch a TED talk that was highly rated, in order to determine heart rate at rest with positive emotional engagement;
- Participants were asked to find a topic for discussion that they could predict would be boring to all participants: participants chose to discuss the topic “what are the most effective gardening strategies?” as a topic for discussion they anticipated would be boring, to determine heart rate while speaking but with low emotional engagement.

Two activities were designed by the investigators to be engaging and promote “a new approach or way of thinking”: activity:

iv “Knowing what you know about heart failure, come up with an analogy: A failing heart is like…”;
v “How can you tell if a fellow student really knows their stuff…or is faking it?”.

Students completed the self-report after each activity to confirm predicted engagement, and heart rate was measured as the dependent variable in this experiment.

#### Part 3. In-class sessions to assess correlation of task engagement and performance on subsequent assessment (cohort four)

101 students tackled two in-class activities designed to improve understanding of the cardiac cycle and completed the engagement self-report for each task. The following week, students completed a written assessment task, involving analysis of how a disease state they had not seen before would affect the cardiac cycle.

### Self-report instrument construction

The engagement self-report was designed to allow completion within one minute by an experienced user, so as to avoid impacts on the learner’s ability to engage with the class. Pairs of items within the six-item report were chosen to address an engagement component (emotional, behavioural and cognitive); these were taken from validated engagement instruments as shown in Table 2. The items were rated on a five point Likert Scale ranging from strongly agree to strongly disagree. In order to determine the validity of the six items as a measure of engagement, Principal Component Analysis was constructed using the data from the 123 participants. Kaiser-Meyer-Olkin Measure of Sampling Adequacy was 0.84, and Bartlett’s test of Sphericity was significant (0.000).

**Table 2:** Self-report items and source.

Activity name: ______________ During this activity:	Item Source	Engagement component
1. I devoted my full attention	Schools engagement measure SEM	Behavioural
2. I pretended to participate	Engagement vs disaffection with learning, EvsD	
3. I enjoyed learning new things	Engagement vs disaffection with learning, EvsD	Emotional
4. I felt discouraged		
5. The activities really helped my learning	Motivated strategies for Learning MSLQ	Cognitive
6. I tried a new approach or way of thinking about the content	New	

For cohorts one and two: The survey was completed on paper every ten minutes, which sometimes aligned with a different in-class activity, when compared to the class activity log. For cohorts three and four, students wrote down the name of the activity they were rating during each ten-minute period.

Reliability of the six-item self-report was assessed using Cronbach’s alpha, calculated for each cohort and for the pooled data, using SPSS. The six items taken together as a measure of engagement showed strong reliability, with Cronbach’s alpha values of 0.70, 0.81, 0.78 and 0.77 for each cohort respectively, and 0.79 for the pooled data. The engagement score for each item was calculated by adding the score for the positively phrased items (1,3,5,6) and subtracting the score for the negatively phrased items (2,4).

### Participant heart rate measurement

Participants were provided with a wrist-band heart rate monitor (Mio Alpha 2) for the duration of the study. Participants were asked to begin measurement 2 minutes prior to the beginning of each class. Heart rates were monitored continuously during the class. At the completion of the class, heart rates were analysed: individual heart rates were sampled every minute and averaged for the individual and for the group present at that class (group) for each ten minute interval.

### Detailed student engagement observation protocol

#### Cohorts one and two were rated by a trained observer using a published method

Students were rated as engaged or not engaged every ten minutes by a single trained observer for cohorts one and two, using an adaptation of the protocol published by Lane and Harris (15). We added the behavior ‘unresponsive / zoned out’ to the list of disengaged behaviours. Engaged behaviours included i) listening / reading; ii) engaged instructor interaction; iii) engaged typing / writing; iv) engaged student interaction. Disengaged behaviours included i) off task activity; ii) settling in or packing up; iii) unresponsive "zoned out"; iv) disengaged technology use; v) disengaged student interaction / distraction.

#### Rating interval reduced and two observers used for cohort three

For the third cohort, having recognized that there was considerable variability in observed engagement within each ten minute interval, students were rated at 2.5 minute intervals by three trained observers; PW, KF and MW. Inter-rater reliability for the categorization of engaged versus disengaged was high; kappa = 0.72, 0.76 and 0.86 for the characterisation of each student as engaged or not engaged during each ten-minute interval. Inter-rater reliability for rating the sub-categories of engagement or disengagement during each period was poor, kappa <0.4, and therefore no further analysis was performed using this data.

### Determination of activity type and Bloom’s level

Each of the 105 activities for cohorts 1–3 was evaluated for the level of active learning within the activity by three independent raters, PW, NK and SS, using the lecture capture recording. 22 activities were categorized as didactic, (recording: instructor speaking and the slide showing content rather than an activity). 31 activities were rated as socratic (recording: instructor leading a whole class discussion, activity slide visible). 25 activities were rated as small group open problems (recording: student conversation hum audible, task on slide with no single correct answer). Finally, 28 activities were rated as small group closed problems (recording: student conversation hum audible, task on slide with single correct answer). The inter-rater reliability for raters one and two was high, kappa = 0.80; Inter-rater reliability for raters one and three was moderate, kappa = 0.58. Overall there was 82% agreement

### Focus groups

The focus groups were conducted at the conclusion of the study in order to determine participant perceptions of the engagement self-report instrument, in particular how they perceived each item and how confident they were in responding to each item. Three separate focus groups were conducted for the 22 students in cohorts 1–3. Each focus group was conducted using a semi-structured approach, involving the same questions being posed to each group prior to free discussion. The questions related to i) the student perceptions of the meaning of each self-report item, and how they approached their ratings to that item; ii) whether the self-report interfered with their learning and iii) whether the items captured their engagement. The conversations were recorded and transcribed.

Coding of the transcripts into themes was performed by two of the authors (PW and NK), using NVivo (QSR International, Cambridge, MA). In the first round, the text was coded into the following pre-determined nodes: six nodes for content related to each of the self-report items 1–6; interference with learning; confidence with ratings; validity of self-report. In the second round, a thematic analysis following the principles of Braun and Clarke [23] was used to identify emergent themes within each node. A meeting between the two investigators was held to remove, merge or divide themes to produce a final coding scheme. In a final round, two investigators analysed the three transcripts, using the coding scheme previously developed, and compared results to verify the coding. No new themes or sub-themes were identified in the final round, suggesting that saturation was achieved.

### Statistical analysis

Inter-rater reliability for observer engagement rating and for task Bloom’s level and activity rating was calculated using GraphPad Prism. Cronbach’s alpha values for the self-report item instrument were determined using SPSS. Repeated measures analysis of variance was used to compare responses to the self-report survey items for different activity types and for different instructors, followed by Tukey’s post hoc test for individual comparisons, and was calculated using SPSS. One way analysis of variance was also used to compare heart rates in the controlled experiment (cohort two). Correlational analysis was used to determine whether heart rate was related to student self-reported engagement; Pearson’s r and associated *p* value were determined using SPSS.

## Results

### Student engagement self-report performance

Four student cohorts (n = 9, 8, 5 and 101 respectively) rated 107 activities using the self-report, for a total of 651 individual self-reports (Table 3). Table 3 shows the mean, standard deviation and n values for each of the variables measured in the study. As this study involved repeated measures, the number of discrete data points that was obtained from the n individual participants is shown in parentheses after the n values.

**Table 3:** Mean, standard deviation and n values for the variables measures in this study.

Variable	Mean	S.D	N
1. I devoted my full attention	3.7	1.0	123 (585)
2. I pretended to participate	2.4	1.2	123 (576)
3. I enjoyed learning new things	3.7	0.9	123 (574)
4. I felt discouraged	2.1	1.0	123 (572)
5. The activities really helped my learning	3.8	0.9	123 (564)
6. I tried a new approach or way of thinking about the content	3.1	1.0	123 (531)
Engagement score (self-report aggregate)	6.0	3.6	123 (530)
Heart rate	119	12	22 (496)
Observed engagement (%)	85	15	22 (357)

Principal Component Analysis was constructed with oblique rotation. This analysis revealed one factor with eigenvalues greater than one, and evaluation of the screen plot confirmed that a one-factor solution was the most appropriate. The one factor accounted for 51.6% of the variability in the item set and was labelled engagement score: this factor was used throughout the rest of the study. The self-report engagement score for each activity for each participant was determined by adding the scores for the three pairs of self-report items and using the following calculation: Engagement score = item 1-item 2 + item 3-item 4 + (item 5+Item 6)/2.

The inter-item correlation matrix showed that there were significant correlations between a number of the variables, and that there were not stronger correlations between the items that were included to represent behavioural, emotional and cognitive engagement (i.e. items 1,2; 3,4 and 5,6) than the correlations between items across those three constructs (see Table 4).

**Table 4:** The inter-item correlation matrix for the student engagement self-report.

	Item 1	Item 2	Item 3	Item 4	Item 5	Item 6
**Item 1**	1	0.30	0.49	0.38	0.46	0.36
**Item 2**		1	0.34	0.36	0.35	0.29
**Item 3**			1	0.50	0.66	0.45
**Item 4**				1	0.41	0.32
**Item 5**					1	0.52
**Item 6**						1

### Observed engagement vs self-reported engagement

Of note, students in this study reported that they “pretended to participate” in 23% of all 10-minute intervals (130 out of 575 reports). The average observed group engagement reported for those 130 time points at which individual students reported that they were pretending to engage was 85% -i.e. at those time points at which students reported that they were pretending to participate, 85% of students in the group were reported to be engaged. There was no correlation between student self-reported engagement and observer group engagement (see S1 Fig).

### Heart rate correlates with self-report items one, three and particularly item six, but not at all with observed engagement %

Table 5 shows correlations between heart rate and percentage of students engaged as measured by observation score and each of the items in the self-reported engagement instrument. Both individual heart rate and grouped heart rate showed positive correlations with items three and six, with the strongest correlation being for item six. The percentage of students rated as engaged by two observers showed no correlation with individual self-reported engagement at self-reported engagement for that cohort. S2 Fig shows heart rates for individual students during a single class.

**Table 5:** Correlation of heart rate, change in heart rate and engagement self-report.

	Engagement score	Item 1	Item 2	Item 3	Item 4	Item 5	Item 6
Individual heart rate	0.15**	0.09	-0.07	0.11*	0.10*	0.10*	0.19*
Individual heart rate max-min	0.14*	0.08	0.13*	0.12*	0.07	0.12	0.23**
Individual observation % engaged	0.05	0.07	0.02	0.08	-0.02	0.08	0.02
Group heart rate	0.22*	0.16	0.08	0.19*	-0.13	0.17	0.24*
Group heart rate max-min	0.29**	0.18	-0.21	0.24*	0.14	0.22	0.25*
Group observation % engaged	0.14	0.10	0.09	0.19	-0.02	0.17	0.13

1—I devoted my full attention. 2—I devoted my full attention. 3—I enjoyed learning new things. 4—I felt discouraged. 5—The activities really helped my learning. 6—I tried a new approach or way of thinking about the content.

N = 105 items for group data and n = 652 for individual data. Data shown are Pearson r correlations

* indicates *p* ≤ 0.05.

** indicates *p*<0.01 for the correlations. Note that there was no significant correlation between heart rate, heart rate change and observation % engaged.

### Self-reported engagement was positively associated with small group, open-problem activities

**Fig 1:**
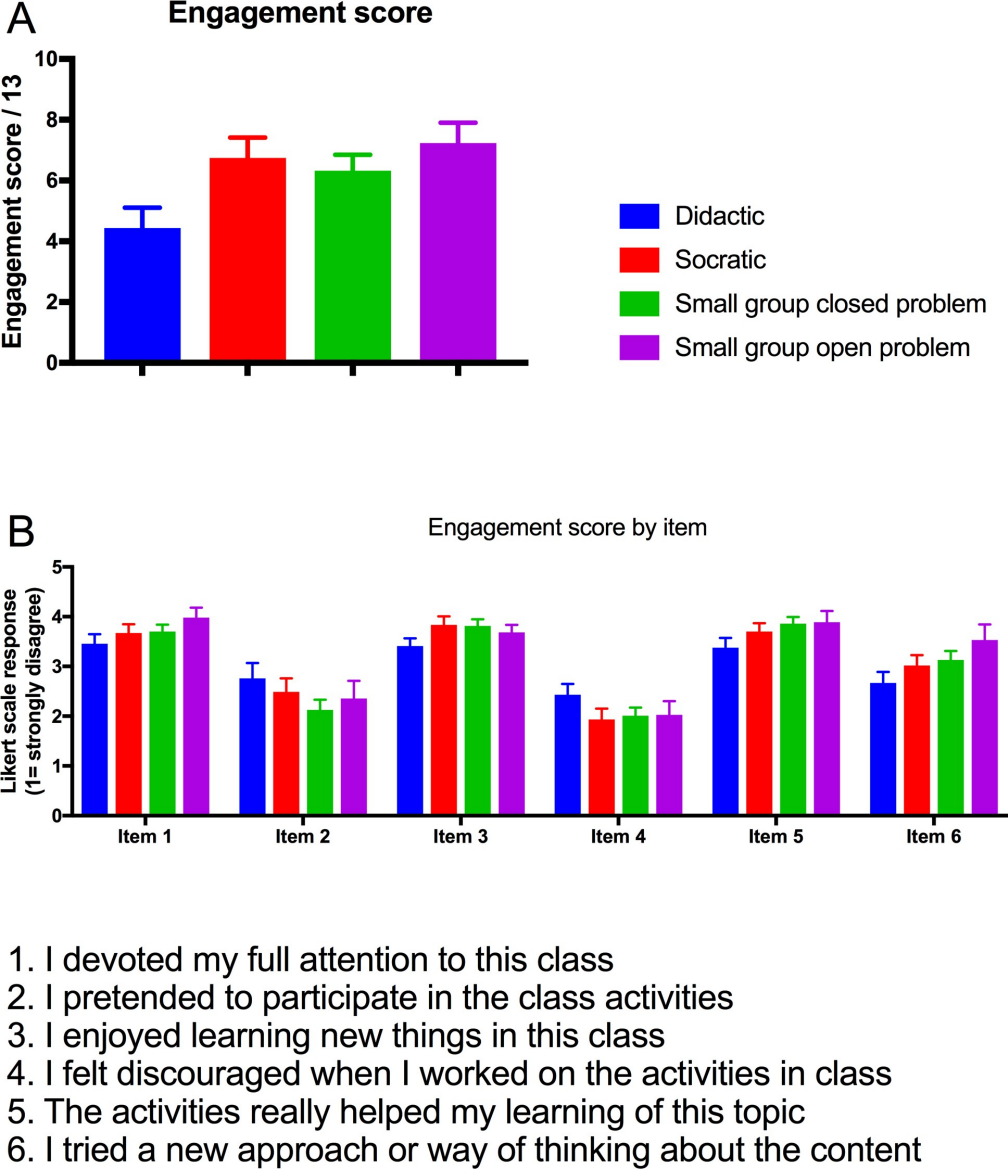
Small group, open problems were associated with higher reported engagement score. Individual engagement score (A) and self-report items (B) were compared for class 10 minute intervals (n = 123). The number of activities rated in each category was as follows: didactic (22), Socratic (31), small group closed problems (28) and small group open problems (25).

A one-way, repeated measures ANOVA was conducted to determine the effect of activity type on student engagement in activities rated as didactic, Socratic, small group closed activities and small group open activities. There was a significant main effect of activity type on student engagement score (ANOVA, F(3, 60) = 3.39, p = 0.02). The mean (SD) engagement scores for the groups were 4.4 (0.7) for didactic activities, 6.7 (0.4) for Socratic activities, 6.3 (0.5) for small group closed activities and 7.2 (0.7) for small group open activities (Fig 1). Post-hoc analysis indicated that responses for the engagement score were significantly higher for activities rated as small group, open tasks than for didactic or Socratic activities (Tukey’s post-hoc test, p<0.05). Whilst there were no significant differences between responses for items 1–6 for small group, closed problems and didactic or Socratic activities, there was a general trend towards greater agreement for small group closed activities for items one, three and six.

### Student self-reported engagement was largely unrelated to Bloom’s level

**Fig 2:**
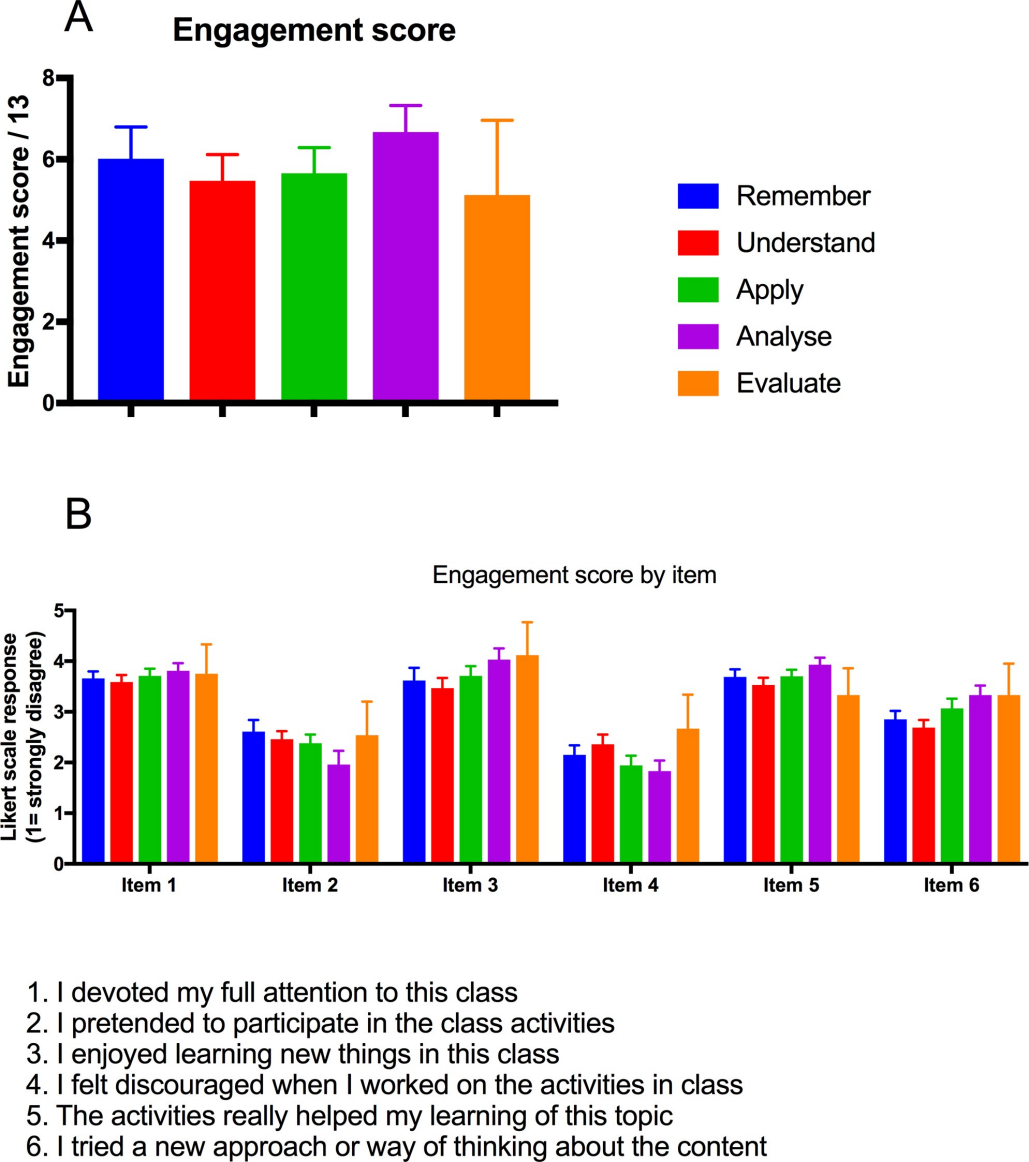
Bloom’s level was largely unrelated to student self-reported engagement. A) Engagement score and B) responses to self-report items was compared for class 10 minute activities classified as remember (8 activities), understand (54 activities), apply (26 activities) analyse (18 activities), or evaluate (4 activities).

A one-way, repeated measures ANOVA was conducted to determine the effect of the Bloom’s level of an activity on student engagement, for activities requiring students to remember, understand, apply, analyse or evaluate. There was no significant effect of Bloom’s level on engagement score for the five Bloom’s levels (F4, 57 = 0.55, *p* = 0.70). The mean (SD) engagement scores for the Bloom’s levels were: Remember: 6.0 (0.8); Understand: 5.5 (0.6); Apply: 5.7 (0.6); Analyse 6.7 (0.7) and Evaluatae 5.1 (1.8) (Fig 2).

### Student self-reported engagement varied between instructors

**Fig 3:**
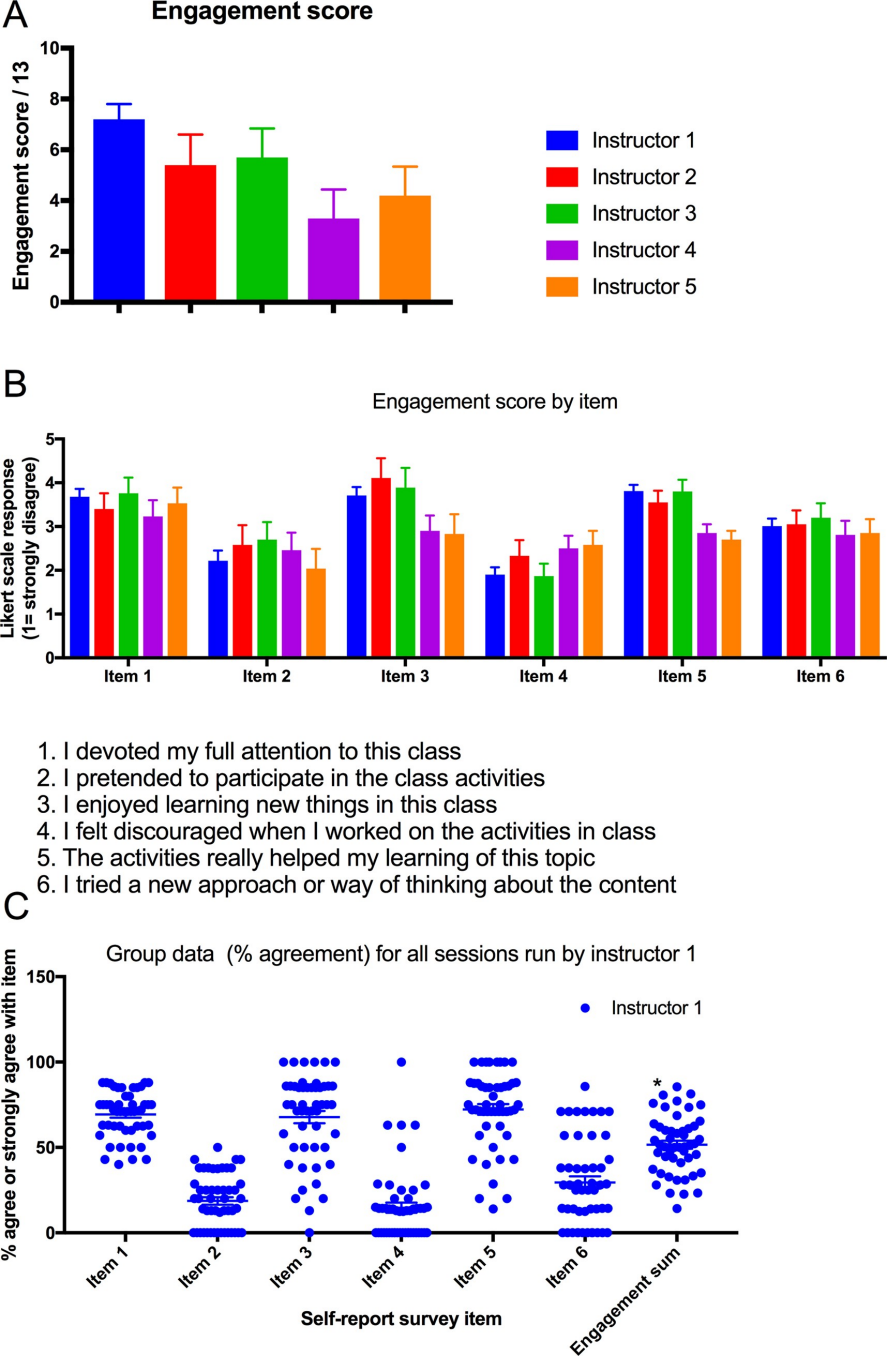
Engagement varied widely between classes from different instructors and between different classes from the same instructor. A) Engagement score and B) self-report scores were compared for the five instructors involved in the study. Bottom panel: engagement also varied widely within the group of classes rated for a single instructor.

A one-way, repeated measures ANOVA was conducted to determine the effect of varying the instructor who led an activity on student engagement, for five individual instructors. There was a significant effect of varying the instructor on engagement score (F(4, 33) = 3.05, *p* = 0.03)). Mean (SD) engagement scores for the instructors were: instructor one 7.2 (0.6); instructor two 5.4 (1.2); instructor three 5.7 (1.1), instructor four 3.3 (1.1) and instructor fivew 4.2 (1.1), see Fig 3.

### Student self-reported engagement was not associated with the positioning of the activity within the class

**Fig 4:**
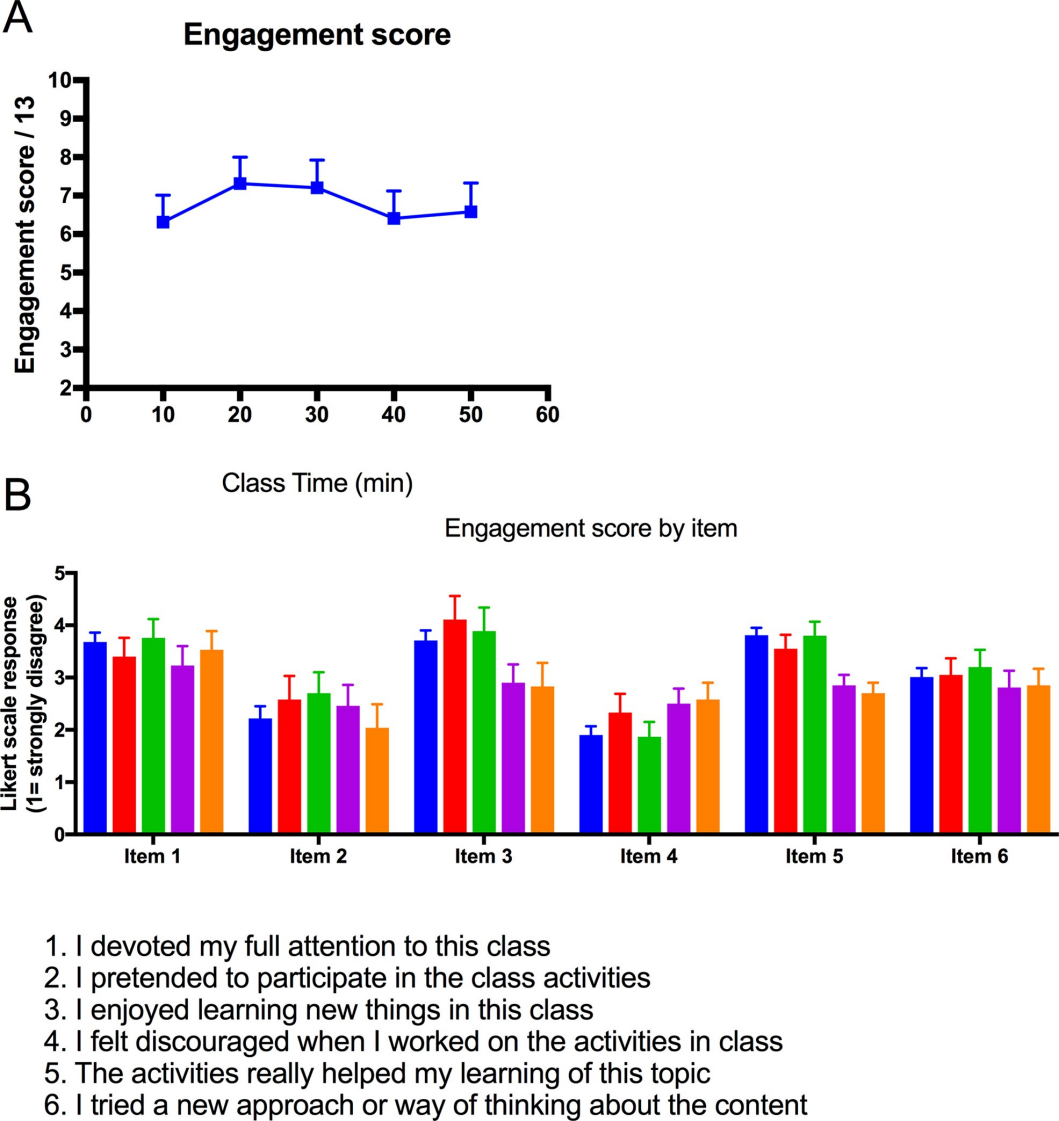
Student self-reported engagement over time during class. A) Engagement score and B) self-report item scores over the 50 minute class time.

A one-way, repeated measures ANOVA was conducted to determine the effect of varying the time during class for an activity on student engagement for five time periods: 0–10, 11–20, 21–30, 31–40 and 41–50 minutes after class began. (Fig 4). There was no significant effect of varying time during class on engagement score (F(4, 76) = 0.43, *p* = 0.03. Mean (SD) engagement scores for the time periods were: 0–10 minutes: 6.3 (0.7); 11–20: 7.3 (0.6); 21–30: 7.2 (0.7); 31–40: 6.4 (0.7) and 41–50: 6.6 (0.8). However, there was a trend towards items one, three, five and six, being positively phrased, showing lower scores in the last ten-minute period, whilst the items phrased negatively (two and four) showed higher scores, on average, in the last ten minutes of class (see Fig 4).

### A controlled experiment showed that student heart rates rise during synthesis tasks, and fall during boring tasks or passively watching videos

**Fig 5:**
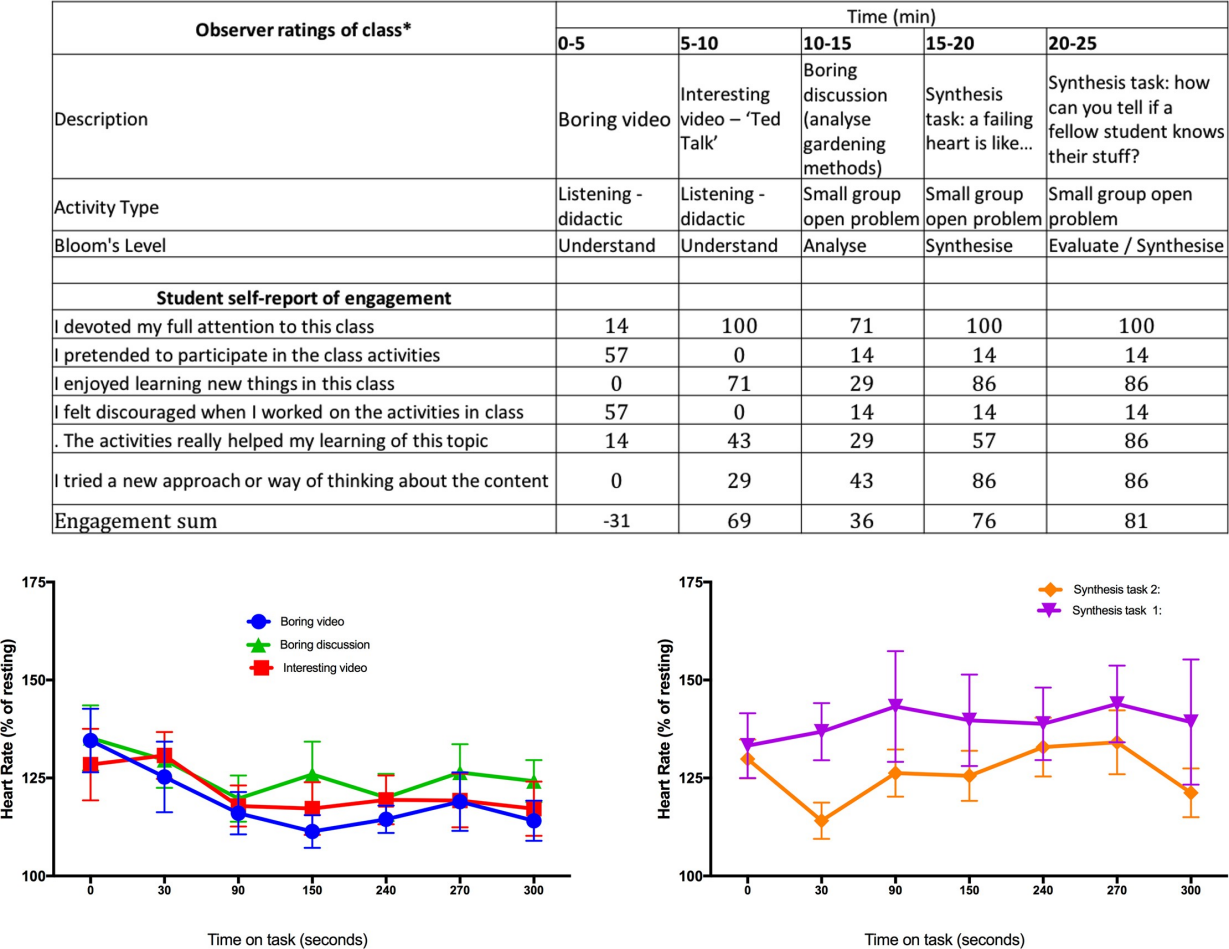
A controlled experiment shows that student heart rates rise during synthesis tasks, and fall during boring tasks or passively watching videos. Students completed the self-report after each activity to confirm predicted engagement; 6 of 7 students agreed with item 6 “I tried a new approach or way of thinking about the content” for task 1 and task 2, whilst zero, two and three of seven students agreed with item 6 for the two videos and boring discussion respectively.

A controlled experiment was conducted to attempt to replicate heart rate increases observed when students agreed with item 6 for novel tasks that required synthesis (Fig 5). A one-way ANOVA was conducted to determine the effect of activity type on heart rate, for two activities during which students “tried a new approach or way of thinking about the content” and three activities during which students did not try “a new approach or way of thinking about the content”. There was a significant effect of varying activity type on heart rate (F(4, 30) = 3.04, *p* = 0.03). Mean heart rates rose by 7.7% of resting heart rate and 1.6% of resting heart rate for the two activities during which students reported trying a new approach or way of thinking about the content (tasks 1 and 2) and fell by 17.9% (9.4), 10.2% (17.0) and 11.9% (15.6) of resting heart rate (SD = 0.4) during the first three minutes of activities during which students did not report trying a new approach or way of thinking about the content (watching a boring video, watching an interesting video and engaging in a boring discussion respectively).

### Student agreement with self-report items one and six for active learning tasks is associated with improved performance on subsequent assessment performance

**Fig 6:**
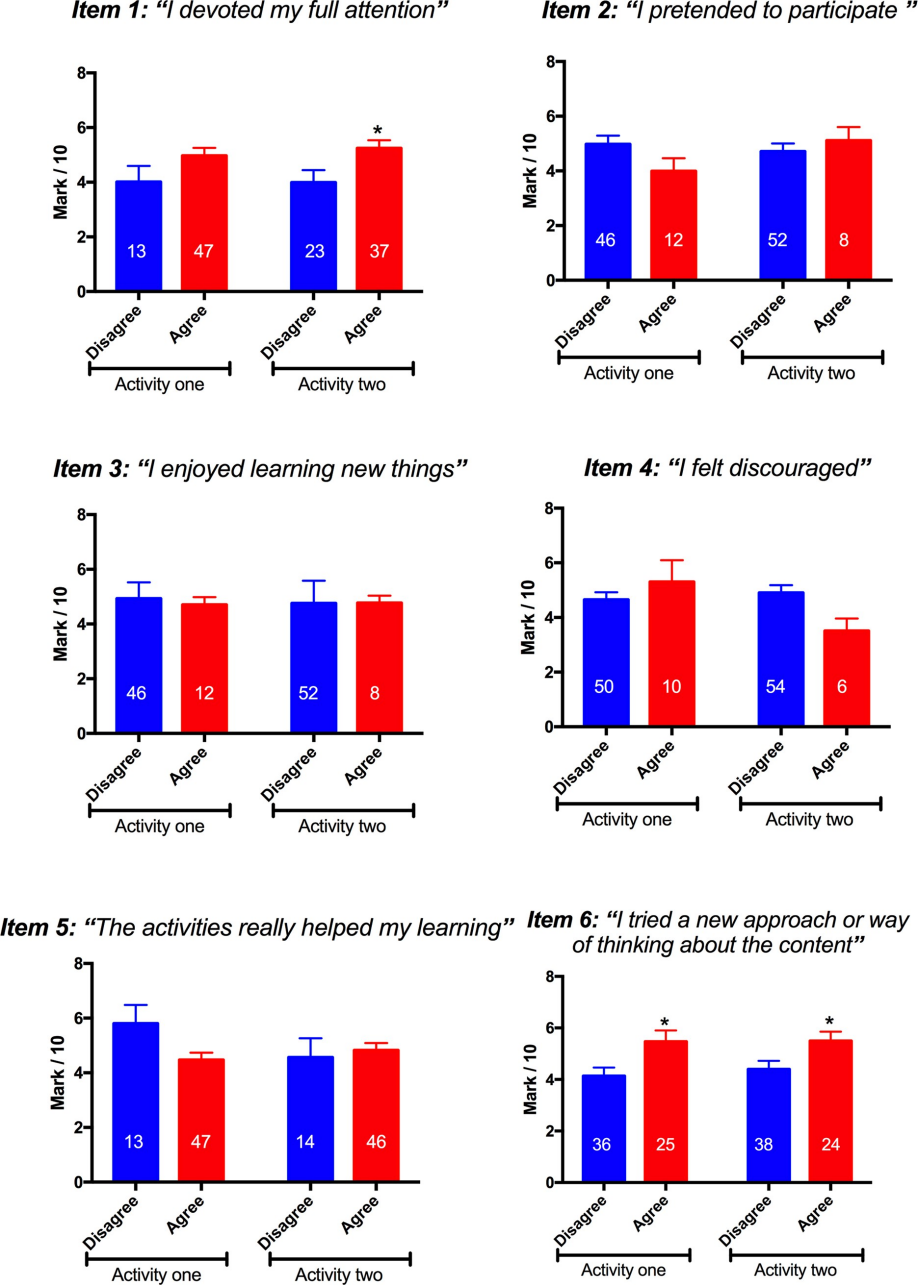
Item agreement vs task performance. Student performance on the assessment task was compared between students who agreed with each self-report item and students who disagreed with that item. N values shown. * indicates *p =* 0.01, *t*-test, n = 23 for item 1 and * indicates *p =* 0.003, *t*-test, n = 25 for item 6.

101 students tackled two activities designed to improve understanding of the cardiac cycle in class and completed the engagement self-report for each task. The following week, students completed a written assessment task, involving an analysis of how a disease state they had not seen before would affect the cardiac cycle. Fig 6 shows the performance of students on those assessment tasks, binned according to whether they agreed with each item of the self-report on not. Students that agreed with items one and six for the active learning activities showed higher performance on the assessment task the following week than students who disagreed with that item. There were no significant differences in assessment performance for the other items between students who agreed and students who disagreed.

### Focus groups

The transcripts of the three focus groups were analysed thematically. Table 6 shows the outcomes of the thematic analysis.

**Table 6:** Themes identified for student perceptions of each of the self-report items, and then for the perceived student confidence in rating and interference with learning. The number of comments (n) for each emergent theme is shown alongside one or two exemplar comments for that theme.

Focus group topic	Item / Theme	n	Exemplar comments
**Item one:**	Paying full attention meant actively thinking and not being distracted	9	*STUDENT*: *Item one meant that I didn't talk to any of my friends*, *I didn't check my phone that I was just sitting there listening*. *STUDENT*: *For me it was also*, *was I really trying to listen*. *Like if we were asked a question or asked to discuss something*, *did I actually try and do it*.* *..*Am I actually thinking about it or am I just sitting there*.
**Item two:**	Pretending to participate involved pretending to listen to the instructor OR pretending to talk about the content	12	*STUDENT*: *It meant that when we were told to discuss with our friends that when he walked past*, *we'd be pretending to talk to each other*. *For me it was also*, *I would classify me pretending to listen if I was sitting there not on my phone*, *not talking to anyone while he was talking but just sitting there*. *I was looking at the content*, *but not really thinking about it*.* *
	There was a difference between pretending to participate and being openly disengaged	9	*STUDENT*: *But with me I wasn't necessarily pretending*, *I was outright talking*.
**Item three:**	Enjoyment occurred during tasks or activities	11	*STUDENT*: *I feel like because we are all here to learn*,* or obviously we are all here to do our best in the course*, *we kind of force ourselves to have a minimum level of attention and engagement in the class*. *But the higher levels of real enjoyment and engagement come from the activities*,* definitely*.
	Enjoyment was associated with task-related learning	18	*STUDENT*: *So I think every time I went to evaluate that I was thinking about if I enjoyed learning about those answers to those case studies*. *So applying my knowledge that was how I enjoyed* *FACILITATOR*: *So*, *enjoyment was understanding*? *STUDENT*: *Yeah*. *STUDENT*: *Yeah that was like me too*.
**Item four:**	Frustration was the most common cause of discouragement	12	*STUDENT*: *When we were discussing things and we didn't know how to start*. *When I didn't know how to approach the question*. *Or when “instructor” comes around to hear what we have to say and we just can't put it in words*, *I see that as discouraged*. *Where I kind of get it but I don't know how to say it*.* *
**Item five:**	Activities that “really helped” resulted from discussing answers in depth	7	*FACILITATOR*: *How did you judge whether what he said or the activities helped you in learning the topic*? *STUDENT*: *Through discussions*. *STUDENT*: *Yes*, *if it was interactive then I would understand it more*. *STUDENT*: *And you have a better judgement of you understand something as well because you are explaining it to your friends*, *or you look at a questions and you know how to answer it*.
**Item six:**	A new approach involved a new understanding of the content	10	*STUDENT*: *I think that's the box I checked agree the least for because I don't know—to reroute the way that you originally learned something and the way you originally thought that something happened and completely change that in a 10 minute segment is a big feat*, *I think*. *And so it was something like where we talked about a drug that wasn't in the pre-class video*, *and I connected it to something in the pre-class video that I understood then*, *I think that's whenever I checked agree for that statement*.
**Did the self-report interfere with learning?**	Not significantly after the initial adjustment	15	*STUDENT*: *Yes*, *I think probably the first lecture*, *I was like what are these questions and it probably took me a bit more time but after that I didn't really feel like it impacted that much*. *I felt it was pretty quick*, *it didn't really hinder my learning*.

## Discussion

### Triangulation of self-report, heart rate and observation as a composite measure of engagement

Our intention of combining student self-report, observation and heart rate to better measure real-time, task-related engagement was unsuccessful, primarily because student self-reported engagement showed minimal correlation with engagement reported by an observer. This lack of correlation is significant in of itself; addressed later in this discussion. There was also no significant correlation between heart rate and observed engagement. There were correlations between heart rate and some of the self-report items, again of interest but not enough to justify the widespread use of measurement of heart rate combined with self-reported engagement. We submit that the use of student self-reported engagement alone can provide educators with useful real-time feedback to guide their active learning activity design and improvement. Specifically, the self-report can identify highly engaging activities that should be re-used and others that require review or discard. However, we note that self-report alone is still limited by the difficulty of recruiting enough students to make for a representative perspective.

### Discrimination, reliability and validity of the student self-report

The self-report showed good reliability. Self-reported engagement was highly variable (means for the six items ranged from 2.1–3.8, S.D. ranged from 0.9–1.2); much more variable than observed engagement levels (mean 85%, S.D. 15), suggesting a greater degree of discrimination between activities for the self-report than the observation score. The ability of the instrument to discriminate between activities was further supported by the high level of variability between activities given by the same instructor (intra-instructor variability). The six self-reported engagement items were all included in the engagement score based on principal component analysis, and showed good reliability as a construct, with Cronbach’s alpha values ranging from 0.70–0.81.

We tested the predictive validity of the self-report using a number of variables that have been shown to be related to engagement in previous studies. A number of studies have shown that teacher behaviours and attributes can influence engagement (see [24]). Voelkl (1995) found that teacher warmth, caring and supportiveness was strongly related to student engagement [25]. We predicted that a valid engagement instrument would show discrimination between instructors and between different activities led by the same instructor. This was found to be the case; inter-instructor variability was seen to contribute more to the variability in engagement between activities than other variables examined (Bloom’s level, activity type, time within class).

We predicted that the type of activity would influence engagement ratings, and specifically, small group, open tasks were predicted to be the most engaging of the range of tasks evaluated, and this proved to be the case. Interestingly, whilst didactic teaching segments were generally rated as less engaging than small group tasks, there was significant variability in the engagement reported for segments classed as didactic. Active learning activities are well established as more engaging than passive learning experiences [2, 5, 26]. Focus group discussions confirmed that this was the case, on the whole; students reported positive task related emotions in each of the focus groups, and the theme “Enjoyment occurred during tasks or activities” was identified as an emergent, unprompted theme during the discussions. However, the focus group analysis also showed task-related frustration as an emergent theme, mostly related to students reaching an impasse, a well characterized phenomenon in motivation research [21, 22, 27].

### Correlation of self-reported engagement with student performance on related tasks

A wide body of literature from K-12 education shows that greater engagement is associated with superior student performance [28, 29] and that disengaged students perform worse [30] on a range of assessments, independent of the variety of methods used to measure engagement. In a nationwide study of 13,000 eighth grade students, student participation during class was strongly related to achievement outcomes [25]. In the current study, the cumulative engagement score did not correlate with their performance on the in-class assessment tasks. However, students that agreed with self-report items one (behavioural engagement) and six (cognitive–I tried a new approach or way of thinking about the content) performed better on assessment tasks than students that disagreed with those items. A future aim of our research is to investigate whether the deliberate design of activities that stimulate new cognitive approaches to a concept–such as asking students to develop their own analogy for a concept–result in improved performance on assessments that test attainment of that concept.

### Pretending to engage

Observation, as a measure of student engagement, is subject to false positive and false negative ratings. Peterson and colleagues, when conducting combined student and teacher engagement rating for mathematics teaching in K-12, reported that some students who were rated by observers as engaged and on-task later self-reported to have been distracted or not thinking about the materials, whilst other students observed to be off-task were of the view that they were engaged with the content [31]. Previous correlations of self-report and teacher report show a stronger correlation between the two measures for behavioural engagement than emotional. The masking of emotions by students has been cited as a reason for the discrepancy between self-report and teacher report of emotional engagement [11, 32]. Students in our study reported that they “pretended to participate” on 23% of all 10-minute time periods, and for the vast majority of those periods, students were rated by independent observers as engaged. When self-reported engagement was matched directly for individuals for cohort three, students who reported pretending to engage were rated as engaged by the observer over 90% of the time. In focus groups, students reported two related motivations for pretending to participate: avoiding disengaged behaviours out of respect for the instructor, and a desire to avoid “getting into trouble” for off-task behavior. These motivations are aligned well with a previous report from a qualitative study of high school student motivations for pretending to engage [33]. The frequency of latent “false appearance of engagement” is of interest not only for the measurement of engagement but also for those teaching academics who use student behavioural cues to measure and drive adjustments to their teaching. Whilst it may be necessary for observers to be trained in psychology and human behavior to optimally estimate engagement, this approach would not be feasible on a large scale and would not address the issue of pretending to engage. This study calls into question the interpretation of engagement measures determined by observation alone.

### Positive emotional engagement, increased heart rate, and “trying a new approach or way of thinking about the content”

Heart rates from students in this study correlated weakly with engagement score, item one (participated), three (enjoyed learning new things) and six (tried a new approach or way of thinking). Agreement with items one and six also correlated with performance on a related in-class assessment. Finally, in the controlled study, participant heart rates rose during activities in which the majority reported trying a new approach or way of thinking about the content. Taken together, our findings suggest that activities that promote novel cognitive approaches to a problem or concept are associated with positive task-related emotions, and importantly, improved performance on assessments testing attainment of that concept. A body of literature has investigated heart rate in the context of engagement and learning. Heart rate variability with each breathing cycle as a result of modulation of vagus nerve activity by higher centres has previously been shown to be related to an individual’s “readiness to engage with the environment” [34, 35]. Heart rate itself has not been directly linked with cognitive or emotional engagement previously, perhaps because of the subtle relationship seen in our study; not all engagement was associated with a rise in heart rate. Our results suggests that further studies of heart rate and heart rate variability in a variety of controlled settings may shed significant light on the relationship between cognitive and emotional engagement and heart rate.

One interesting aspect of this study was the role of oral participation. Frymier et al [36] found that the relationship between oral participation and engagement was weak, whilst student non-verbal attentiveness was positively associated with engagement as measured by Martin’s Motivation Engagement Scale [37]. We found that small group discussion was rated as particularly engaging when the tasks were open tasks (defined as tasks with no single correct answer).

### Interpreting analysis of single items from self-report

In psychometric research, analysis of constructs involves development and use of scales that are the sum of multiple survey items. In the current study, a scale for engagement was developed and validated. We also analysed individual items in this study, as these items may act as indicators that alert us to potentially interesting phenomena. We don’t fully agree with some in the health sciences education world who feel that single items can reliably assess global constructs (see [38]). However, we do see the value in reporting and cautious analysis of single items as bellwethers worthy of further investigation. We note the potential for over-interpretation of these individual items and therefore advise cautious interpretation of the individual item analysis in this study, and we have now focused the results section on the engagement scale that we developed and validated.

## Conclusions

This study characterized a method for assessing student engagement during classes. The student self-report instrument showed good face and predictive validity and strong reliability, and may prove useful to academic teaching staff in evaluating and refining their active learning activities. Independent observation was not found to correlate with student self-report, due in part to student’s pretending to engage who were rated as engaged by an observer. Students agreed with the statement “I pretended to participate in the class activities” a striking 23% of the time. We suggest that teaching staff be informed of this outcome, given that many use student behavioural cues such as nodding / eye contact as indicators of engagement. We further suggest that in-class assessments of engagement using our instrument would improve the effectiveness of active learning approaches, highlighting engaging activities and identifying poorly engaging activities for improvement or discard, and that further investigation of this hypothesis is needed. Heart rate was, for the first time, seen to relate to positive cognitive and emotional engagement and in particular to “trying a new approach or way of thinking about the content”, and a controlled study reproduced this finding during two activities that required students to try a new cognitive approach to a concept.

## Supporting information

S1 Fig Lack of correlation of observed engagement with self-reported engagement. (PNG)

S2 Fig Heart rates during active learning and didactic class periods. (PNG)
