# Supplementary figures and images for: Development of a self-report instrument for measuring in-class student engagement reveals that pretending to engage is a significant unrecognized problem
Source: PLoS One. 2018 Oct 17;13(10):e0205828. doi: 10.1371/journal.pone.0205828 (PMC6192645; doi:10.1371/journal.pone.0205828)

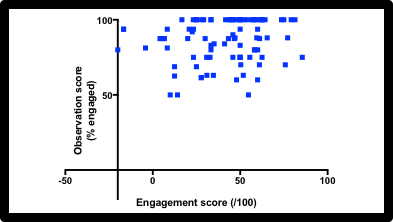

Supplement: S1 Fig — (PNG) [file pone.0205828.s001.png]

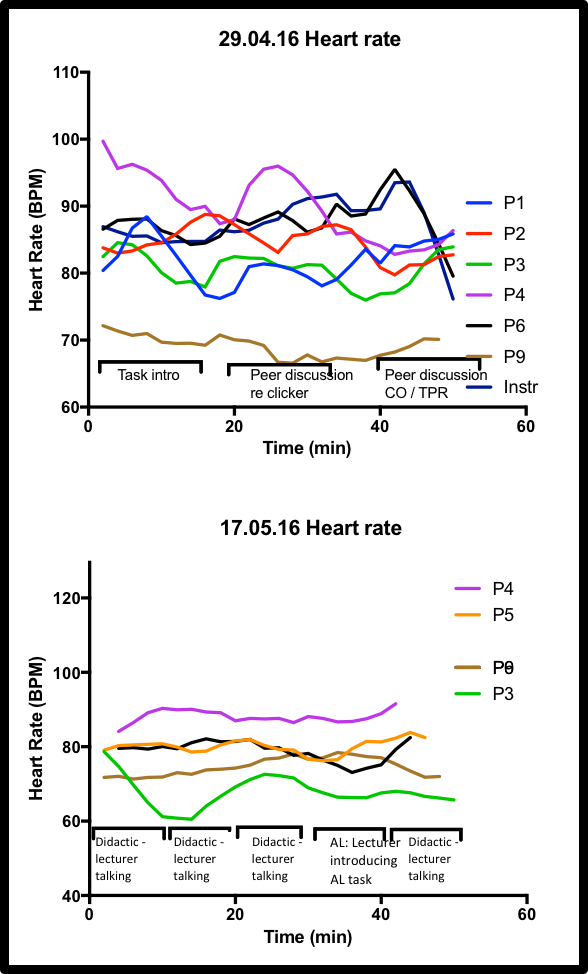

Supplement: S2 Fig — (PNG) [file pone.0205828.s002.png]
